# Confronting Tigecycline-Resistant *Acinetobacter baumannii* via Immunization Against Conserved Resistance Determinants

**DOI:** 10.3389/fmicb.2020.00536

**Published:** 2020-03-31

**Authors:** Ming-Hsien Chiang, Ya-Sung Yang, Jun-Ren Sun, Yung-Chih Wang, Shu-Chen Kuo, Yi-Tzu Lee, Yi-Ping Chuang, Te-Li Chen

**Affiliations:** ^1^Department and Graduate Institute of Biology and Anatomy, National Defense Medical Center, Taipei, Taiwan; ^2^Graduate Institute of Life Sciences, National Defense Medical Center, Taipei, Taiwan; ^3^Division of Infectious Diseases and Tropical Medicine, Department of Internal Medicine, Tri-Service General Hospital, National Defense Medical Center, Taipei, Taiwan; ^4^Institute of Preventive Medicine, National Defense Medical Center, Taipei, Taiwan; ^5^National Institute of Infectious Diseases and Vaccinology, National Health Research Institutes, Zhunan, Taiwan; ^6^School of Medicine, National Yang-Ming University, Taipei, Taiwan; ^7^Department of Emergency Medicine, Taipei Veterans General Hospital, Taipei, Taiwan; ^8^Department and Graduate Institute of Microbiology and Immunology, National Defense Medical Center, Taipei, Taiwan; ^9^Institute of Clinical Medicine, National Yang-Ming University, Taipei, Taiwan

**Keywords:** immunization, resistant determinant, *Acinetobacter*, tigecycline, efflux pump

## Abstract

Antimicrobial-resistant (AMR) bacterial infections, including those caused by *Acinetobacter baumannii*, have emerged as a clinical crisis worldwide. Immunization with AMR determinants has been suggested as a novel approach to combat AMR bacteria, but has not been validated. The present study targeted tigecycline (TGC) resistance determinants in *A. baumannii* to test the feasibility of this approach. Using bioinformatic tools, four candidates, AdeA, *Ade*I, AdeK, and TolC, belonging to the resistance-nodulation-division (RND) efflux pump were identified as highly conserved and exposed antigens from 15 *A. baumannii* genomes. Antisera generated from recombinant proteins showed the capability to reserve Hoechst 33342, a substrate of the efflux pump, in bacterial cells. The rTolC antisera had the highest complement-dependent killing and opsonophagocytosis effect compared to the sera from phosphate-buffered saline immunized mice. Among the antisera, anti-rAdeK-specific antisera decreased the minimal inhibitory concentration of TGC in 26.7% of the tested isolates. Immunization with rAdeK significantly potentiated TGC efficacy in treating TGC-resistant *A. baumannii* pneumonia in the murine model. The bacterial load (7.5 × 10^5^ vs. 3.8 × 10^7^, *p* < 0.01) and neutrophil infiltration in the peri-bronchial vasculature region of immunized mice was significantly lower compared to the PBS-immunized mice when TGC was administrated concomitantly. Collectively, these results suggest that active immunization against resistance determinants might be a feasible approach to combat multidrug-resistant pathogens in high risk population.

## Introduction

Antimicrobial-resistant (AMR) bacterial infections have emerged as a serious problem in clinical settings worldwide. By 2050, 10 million people may die annually from AMR infections (Jansen et al., 2018). Unfortunately, the launch of new antibiotics is rapidly compromised by the emergence of resistance (Alekshun and Levy, 2007). Therefore, the development of new strategies to combat AMR bacteria is urgently needed.

Recently, vaccine development has been increasingly advocated as a new solution to AMR bacteria (McConnell et al., 2011; Huang et al., 2014; Jansen et al., 2018), especially for those with high risk of acquired infection (Gagneux-Brunon et al., 2018). Vaccine candidates often target capsule polysaccharides and virulent factors that are responsible for disease pathogenesis (Rappuoli et al., 2019). Unfortunately, AMR bacteria-targeting vaccines have not been very successful until now due to the heterogeneity in the expression levels of vaccine antigens in different circulating strains of the target pathogen (Garcia-Quintanilla et al., 2016; Rappuoli et al., 2019). Moreover, the immunity induced by single or multiple antigens, which is usually obtained both *in vitro* or *in vivo*, might be ineffective in protecting the host because the pathobiology in human infection is more complicated and remains obscure (Perez and Bonomo, 2014).

Antibiotic resistance determinants could be considered as potential vaccine candidates (Ni et al., 2017). Although there are variations in these resistance determinants in clinical isolates, this vaccination strategy might still be effective in eradicating resistant strains by means of replacing these strains which have reduced fitness with susceptible strains when vaccination coverage is high (Joice and Lipsitch, 2013; Niewiadomska et al., 2019). This approach has an advantage when the resistance determinants are consistently present *in vitro* and *in vivo* and also possess less selection pressure on bacteria (Niewiadomska et al., 2019). However, this approach has not been validated yet.

The present study tested this idea in *Acinetobacter baumannii*. *A. baumannii* is considered one of the most problematic bacteria by the Infectious Disease Society of America (IDSA) (Boucher et al., 2009) because of the rapid evolution of multidrug and pan-drug resistant strains. Currently, only few strains of this bacteria are still susceptible to last-line antibiotics such as, carbapenem and tigecycline (TGC) (Sun et al., 2013; Ni et al., 2016). In order to maximize the coverage of immunization, the antibiotic resistance determinants used for the vaccine candidate should be the major resistance mechanism of the antibiotic of interest in particular bacterial species. These determinants should be universal in different strains, conserved in sequence homology, and accessible by the immune system. In this aspect, carbapenem resistance determinants are not suitable vaccine candidates, as its major resistance mechanism is production of different classes of carbapenemases (Nordmann and Poirel, 2019), which are very diverse in their protein sequences and structures. On the contrary, the major mechanism for TGC resistance is overexpression of efflux pumps (Sugawara and Nikaido, 2014). These are universal and are conserved in *A. baumannii* strains (Ardehali et al., 2019), and therefore, might be good vaccine candidates to test this immunization approach. The present study utilized bioinformatics tools to identify conserved and surface-exposed antigens of the chromosomal-encoded resistome of *A. baumannii*. Several protein components of the resistance-nodulation-division (RND) efflux system were identified, which have been associated with TGC resistance in *A. baumannii*. We propose an immunization approach using TGC resistance determinants in a murine pneumonia model to combat multidrug-resistant *A. baumannii*.

## Materials And Methods

### Bioinformatic Tools

Microbial Genome Database (MBGD) was used for comparative analysis of completely sequenced microbial genomes to identify core genes that are universal from 15 *A. baumannii* genomes (Supplementary Table S1; Uchiyama et al., 2014). PSORTb 3.0.2 (Yu et al., 2010), CELLO2GO (Yu et al., 2014), or SOSUI-GramN (Imai et al., 2008) were applied to predict the conserved residues and sub-cellular localization of these proteins. Comprehensive Antibiotic Resistance Database (CARD) was used to predict the resistome from raw genome sequence using Resistance Gene Identifier (RGI) software (Jia et al., 2017).

### Bacterial Strain Preparation

*A. baumannii* ATCC17978 reference strain was purchased from the American Type Culture Collection (ATCC). TGC-resistant clinical *A. baumannii* isolates were obtained from Tri-Service General Hospital in Taiwan (Sun et al., 2014). All isolates were identified using conventional biochemical and genomic methods as previously described (Sun et al., 2014).

### Construction and Purification of Antigens

Recombinant AdeA (A1S_1751), *Ade*I (A1S_2735), AdeK (A1S_2737), and TolC (A1S_0255) from ATCC17978 were amplified (the primers are listed in Supplementary Table S2) and cloned into a pET-29a expression vector (Vovagen, Darmstadt, Germany) with a 6× polyhistidine tag fused to the C-terminus of the recombinant protein. The resulting plasmids were expressed and purified as described in our previous study (Chiang et al., 2015). Purified proteins were digested with trypsin for subsequent liquid chromatography–mass spectrometry/mass spectrometry (LC-MS/MS) analysis and protein identification was conducted by Mission Biotech Co., Ltd., Taiwan. Sequence similarity comparison of *adeA*, *adeI*, *adeK*, and *tolC* from ATCC17978 and all isolates were analyzed using MEGA7 (Kumar et al., 2016).

### Mouse Immunogenicity Assessment and Pneumonia Models

All animal studies were approved by the National Defense Medical Center Institutional Animal Care and Use Committee (NDMC IACUC-17-206). Female C57BL/6 mice (6 weeks old) were bred in a barrier facility under specific pathogen-free conditions. C57BL/6 mice (*n* = 10/group) were subcutaneously (sc.) immunized with 10 μg of individual recombinant antigens formulated with Complete Freund’s Adjuvant/Incomplete Freund’s Adjuvant (CFA/IFA) (Invivogen, Hong Kong), on days 0, 14, and 28. Blood samples were collected before the last immunization and tested against each immunogen. Immunoglobulin G (IgG) antibody titers were determined using antigen-specific enzyme-linked immunosorbent assays (ELISAs).

For conducting efficacy studies, immunized mice were challenged intra-tracheally (IT) on day 42 with a lethal dose [3 × 10^7^ colony-forming units (CFUs)] of mid-log phase AB247 strain mixed with 10% porcine mucin (Sigma-Aldrich, MO, United States). The use of porcine mucin is to enhance the infectivity of *A. baumannii* (McConnell et al., 2011). TGC (10 mg/kg/d, q12h., sc.) treatment regimen was adopted from that used in a previous study (Pichardo et al., 2010). After 24 h therapy, the blood, lung, spleen, and kidney were homogenized and plated to evaluate for the CFUs. For histological analysis, the excised lungs were placed in vials containing 4% formaldehyde. The lungs were placed under vacuum overnight, paraffin-embedded, and stained with hematoxylin and eosin (HE). Histological scores were assigned by independent pathologists by evaluating 3–5 fields, according to the following criteria (Noto et al., 2017): 0, no pathology; 1, minimal infiltrates of neutrophils in alveolar spaces; 2, low numbers of neutrophils in alveoli; 3, moderate numbers of neutrophils and hemorrhage in alveoli with occasional lobar involvement and focal necrosis of alveolar-wall neutrophils in bronchioles; 4, marked numbers of neutrophils, consolidation, and widespread alveolar necrosis.

### Flow Cytometry

ATCC17978 and clinical isolates from late-log-phase growth (OD_600_ ≈ 1.8) in Luria-Bertani (LB) cultures were diluted in PBS containing 0.5% (w/v) bovine serum albumin (BSA) as a blocking buffer to an OD_600_ of 0.03. Each specific antiserum was added at a 1:100 dilution with the bacterium. Unbound antibody was removed by washing twice, then fixed by incubating with 4% formaldehyde/PBS for 10 min on ice. A secondary antibody, goat anti-mouse IgG-PE (Invitrogen Corp., Carlsbad, CA, United States) at 0.1 mL per well, was added at a 1:100 dilution and incubated for 30 min. The bacteria were analyzed using a FACSCalibur flow cytometer (BD, Franklin Lakes, NJ, United States). Wash buffer, secondary antibody, or PBS Immunize serum were used as negative controls.

### Hoechst 33342 (H33342) Accumulation Assay

H33342 accumulation assays were carried out as described by Richmond et al. (2013). Strains were grown to an OD_600_ of 0.4 and resuspended in PBS at room temperature, and the suspension was adjusted to an OD_600_ of 0.1. Centrifugation steps were carried out at 2500 g. The wells of a black microtiter plate (Corning, Amsterdam, Netherlands) were inoculated with 176 μL of bacterial suspension and 20 μL of 10 μg/mL H33342. After 5 min equilibration, 4 μL of the efflux pump inhibitor, phenylalanine-arginine-β-naphthylamide (PAβN), or specific antisera were added. The fluorescence intensity was recorded every 1 min for 60 min on a SPECTRAmax5 fluorometer (Molecular Devices, Sunnyvale, CA, United States) at excitation and emission wavelengths of 355 and 460 nm, respectively.

### Complement and Opsonophagocytosis Bactericidal Assays

ATCC17978 was freshly grown to a final bacterial cell concentration of 10^6^ CFU/mL, and aliquoted into 96 well microtiter plates (10 μL, 10^4^ cells/well). For complementary studies, 10 μL heat-inactivated or immune sera were mixed with 80 μL of undiluted human complement and added to the wells for 1 h at 37°C. The samples were plated for bacterial enumeration. Bacteriolysis activity was defined as [1 − (CFU immune sera at 60 min/CFU of PBS-immunized antisera at 60 min)] × 100.

For the opsonophagocytic kill assay, RAW 264.7 macrophages were cultured in RPMI 1640 (Irvine Scientific, Santa Ana, CA, United States) with 10% fetal bovine serum (FBS), 1% penicillin/streptomycin, and glutamine (Gemini BioProducts), and 50 μM β-mercaptoethanol (Sigma-Aldrich, St. Louis, MO, United States). RAW 264.7 cells were stimulated with 100 nM PMA (Sigma-Aldrich) for 3 days. RAW 264.7 macrophages (2 × 10^5^/well) and ATCC17978 (1 × 10^4^CFU/well) were added into the wells along with the heat-inactivated or immune sera (5%). After a 1 h incubation with gentle shaking, the samples were serially diluted and plated. Serum killing rates were counted by comparing the number of reduced CFUs with those observed using PBS-immunized antisera.

### RNA Isolation and Quantitative Reverse Transcriptase – PCR

The expression level of *adeA*, *adeI*, *adeK*, and *tolC* were measured by quantitative real-time polymerase chain reaction (PCR) assays as described previously (Rosenfeld et al., 2012). The mRNA expression of *rpoB* from ATCC17978 was used for normalization with specific primers (Supplementary Table S2). The relative gene expression was expressed as fold-change calculated by the ΔΔCt method. Gene expression levels ≥ 2-fold compared to that for the reference strain, ATCC17978, were considered significant overexpression. Each experiment was performed in duplicates and at least twice independently.

### Minimal Inhibitory Concentration (MIC) Determination by Broth Microdilution Method

The MICs of TGC were determined by broth microdilution methods in Mueller Hilton broth and interpreted according to the Clinical and Laboratory Standards Institute guidelines (CLSI, 2017). Since MIC breakpoints are not established for TGC in *Acinetobacter* spp., we used the Food and Drug Administration breakpoints set for *Enterobacteriaceae* (Pillar et al., 2008). Each experiment was performed in triplicate.

### Statistical Analysis

Statistical analyses were performed using GraphPad Prism 7 software. All graphical values were represented as means ± standard error of the mean (SEM). Tests of statistical significance were performed using one-way analysis of variance and Kruskal–Wallis tests with *post hoc* analysis. Differences were considered significant for *p* < 0.05.

## Results

### *In silico* Screening of Conserved Outer Membrane Efflux Pump Proteins and Generation of Recombinant Proteins

**FIGURE 1:**
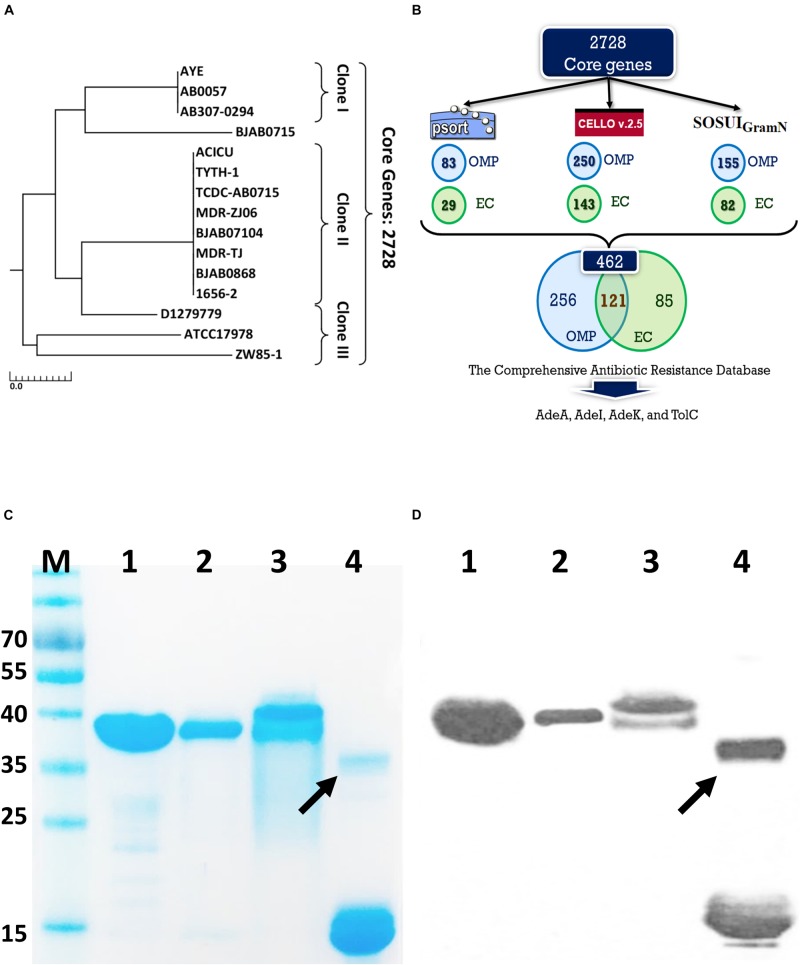
A rational strategy for potential antigen identification and verification from *A. baumannii* genomes. **(A)** The phylogenetic lineage of 15 completely sequenced *A. baumannii* strains analyzed in this study was constructed by the neighbor-joining method performed in MEGA7. Homology core genes were analyzed using the web-based MBGD tool. **(B)** Bioinformatics tools including PSORTb 3.0.2, CELLO2GO, and SOSUI-GramN were utilized to identify 462 non-redundant outer membrane or extracellular proteins from 2728 core genes. Four antimicrobial-resistant genes associated with efflux pumps were identified in the 462-protein datasets based on Comprehensive Antibiotic Resistance Database (CARD) analysis. OMP, outer membrane protein; EC, extracellular protein. **(C)** Purified proteins were analyzed by 12% sodium dodecyl sulfate-polyacrylamide gel electrophoresis. Lane M-standard protein markers, Lane 1-purified rAdeI protein, Lane 2-purified rTolC protein, Lane 3-purified rAdeK, Lane 4-purified rAdeA protein. **(D)** Western blot analysis of purified recombinant proteins using mouse anti-His antibody. The arrow indicates dimerization of rAdeA (confirmed by LC-MS/MS).

Fifteen *A. baumannii* genomes included 12 multidrug resistant strains were used for analysis. The antibiotic susceptibility profiles of these isolates are listed in Supplementary Table S1. These strains were grouped by multilocus sequence typing (MLST) (Laure et al., 2010) and three strains belonged to international clone I (IC I), eight to IC II, three to IC III, and the last strain was unclassified, indicating a wide coverage of *A. baumannii* strains (Figure 1A). A total of 2728 core genes were identified; among them, 462 non-redundant proteins were predicted to be outer membrane or extracellular proteins. CARD predicted that among the 462 conserved and surface-exposed proteins, four were associated with antibiotic resistance, including AdeA (A1S_1751), *Ade*I (A1S_2735), AdeK (A1S_2737), and TolC (A1S_0255) and were selected as vaccine candidates (Figure 1B). All the proteins belonged to the chromosomally encoded RND efflux pump family and had been associated with resistance to multiple antibiotics, including TGC (Sun et al., 2013). The four recombinant proteins were expressed and purified (Figure 1C). The proteins were identified and confirmed by immunoblotting (Figure 1D) and LC-MS/MS analysis (Table 1).

**TABLE 1:** Confirmation of the purified recombinant proteins by liquid chromatography–mass spectrometry/mass spectrometry (LC-MS/MS).

**Sample name**	**Protein name**	**Accession number**	**pI**	**MW (KDa)**	**Sequence coverage**
AdeA	Multidrug RND transporter [*A. baumannii*]	gi| 500174963	5.46	15.714	76%
AdeI	Multidrug RND transporter [*A. baumannii*]	gi| 500185983	9.04	39.778	83%
AdeK	adeC/adeK/oprM family multidrug efflux complex outer membrane factor [*A. baumannii*]	gi| 691007736	9.12	52.770	83%
TolC	RND transporter [*A. baumannii*]	gi| 500183799	9.21	44.273	78%

### Polyclonal Antibody Production and Functional Analysis

**FIGURE 2:**
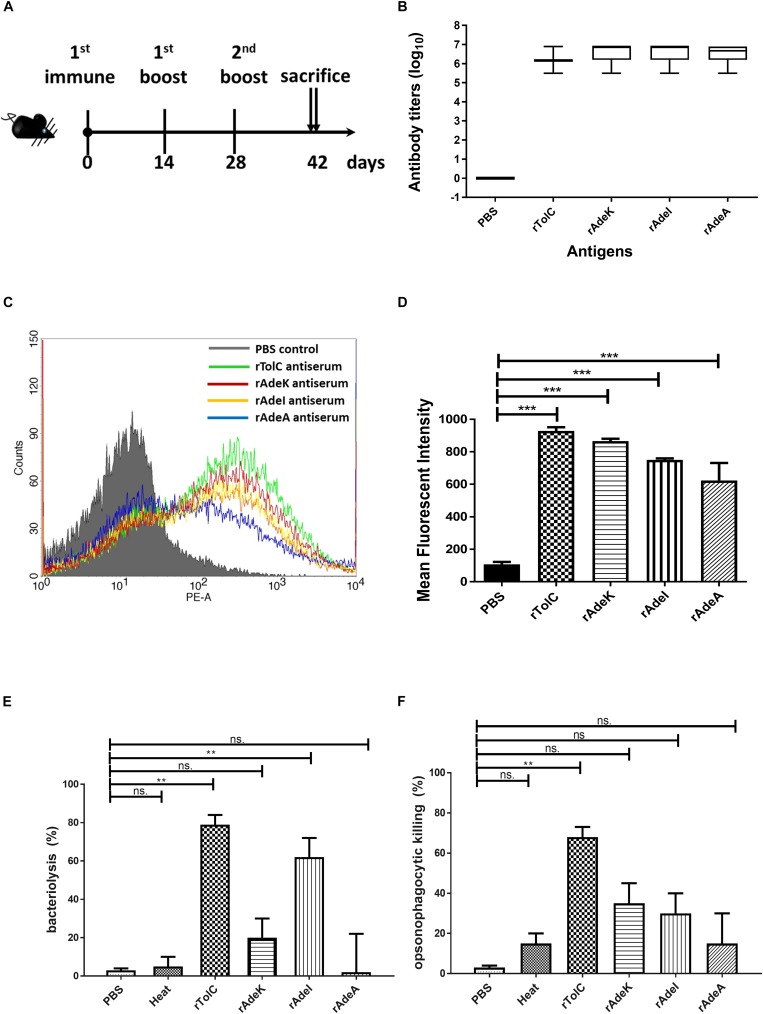
Polyclonal mouse antisera production and characterization. **(A)** Groups of C57BL/6 mice (*n* = 10) were subcutaneously immunized with either 10 μg of individual antigen (rAdeI, rTolC, rAdeK, and rAdeA) or phosphate-buffered saline (PBS) formulated with Complete Freund’s Adjuvant/Incomplete Freund’s Adjuvant (CFA/IFA) on days 0, 14, and 28. Blood samples were collected before sacrifice and tested against each antigen. **(B)** Serum IgG antibody titers against each antigen as indicated by enzyme-linked immunosorbent assay. **(C)** Flow cytometry (FACS) analysis demonstrating the surface accessibility of the antigen-specific antisera. Cells from late-log-phase cultures were probed with a control non-specific antibody (shaded histograms) or individual antisera (unshaded histograms). Each histogram shows the fluorescence intensity distribution of >20,000 flow cytometry events. **(D)** Histogram representing quantitative analysis of the binding intensity from FACS studies. Bars indicate the means of at least three independent experiments ± SEM. ***p < 0.001. **(E)** Bacteria inhibition by each antigen-specific mouse antisera at 1:10 dilution, used in the presence of human complement and ATCC17978. **(F)** The complement-dependent opsonophagocytosis assays were performed with mouse antisera (1:10), ATCC17978 and RAW 264.7 macrophages. The results represent the percentage of bacterial survival in the assay after 1 h of incubation at 37°C compared to that in phosphate-buffered saline (PBS)-Immunized antisera. Bars indicate the means of at least three independent experiments ± SEM. ns., non-significance. **p < 0.01, Heat, complement replaced with a heat-inactivated complement.

We then produced antigen-specific polyclonal antibodies from C57BL/6 mice using immunization (Figure 2A). All recombinant antigens (rTolC, rAdeK, rAdeI, rAdeA) formulated with CFA/IFA induced strong antigen-specific IgG antibody responses (IgG titers > 10^5^, Figure 2B) on day 42 after immunization. The results indicated that all four antigens are highly immunogenic. Antigen-specific antisera were used to verify the location of these proteins in the outer surface of bacteria by flow cytometry (Figure 2C). Data confirmed that all the antisera could bind on the surface of ATCC17978, with significantly higher intensities than that for the PBS control antisera (Figure 2D). *In vitro* complement-dependent and opsonophagocytosis bactericidal assays were used to assess the potential bacteria-killing activity of each antiserum. The results showed that 2–79% of ATCC17978 were inhibited by these antisera (Figures 2E,F). Among them, rTolC-specific antisera had the highest killing efficacy compared to the sera from PBS-immunized mice.

**FIGURE 3:**
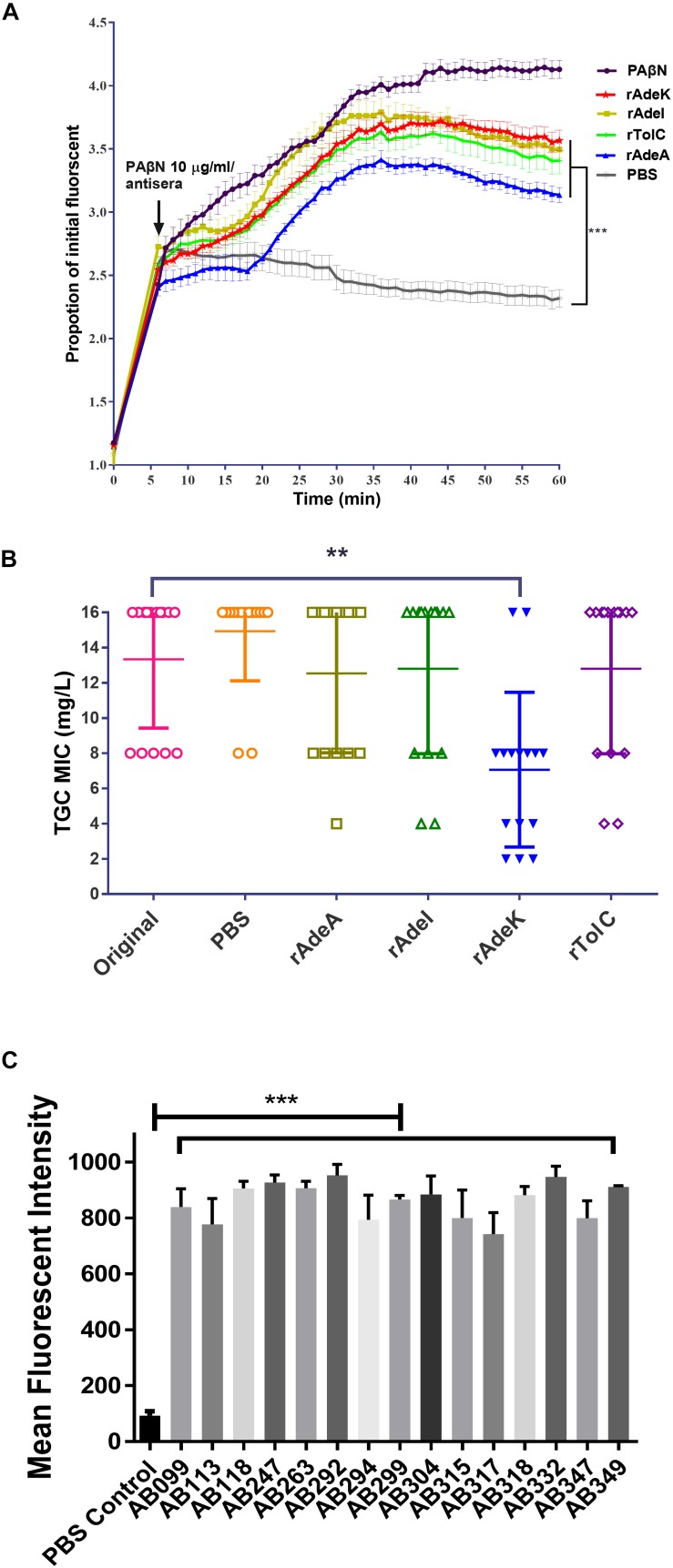
The function of efflux pump inhibition activity of each antiserum. **(A)** Fold changed-H33342 fluorescence values for ATCC17978 following growth in specific antisera and treatment with phenylalanine-arginine beta-naphthylamide (PAβN) (10 μg/mL). Mock-treated cells are also shown (gray line). The assays were performed in triplicates and the error bars show the SEM at 1-min intervals. **(B)** Minimum inhibitory concentration (MIC) of tigecycline-resistant (TGC-R) *A. baumannii* clinical isolates (*n* = 15) compared to original or specific antisera [phosphate-buffered saline (PBS), rAdeA, rAdeI, rAdeK, and rTolC] co-administrated by broth microdilution method. The experiments were performed in triplicates. The *p*-value was determined by Kruskal–Wallis test with Dunn’s multiple comparison analysis. **p < 0.01. **(C)** Histogram representing quantitative analysis of the binding intensity between anti-AdeK antisera and isolates from the FACS study. The bars indicate the means of at least three independent experiments ± SEM. ***p < 0.001.

The accumulation of bis-benzamide H33342 dye provides a reliable method to evaluate the effect of agents that can block efflux pumps (Richmond et al., 2013). The fold-change of fluorescence intensity dramatically increased after adding PAβN and specific antisera, except for the PBS antisera control (Figure 3A, *p* < 0.001). Notably, fluorescence intensities were slightly decreased after 40 min of antisera treatment.

To examine the synergistic effect of antisera in the MICs range for TGCs in *A. baumannii*, 15 clinical TGC-R *A. baumannii* isolates were tested (Table 2). The TGC MICs of all isolates were > 4 mg/L. The sequence similarities of *adeA*, *adeI*, *adeK*, and *tolC* were determined (Table 3) and the similarities were very high among the isolates (98.2–100%), compared to the ATCC17978 sequence. The quantitative expression levels of *adeA, adeI, adeK*, and *tolC* were also determined. In general, all strains showed more than one pump overexpression phenotype. TGC MICs were significantly reduced after a combination with rAdeK antisera was used (Figure 3B), and five of the tested isolates (AB099, AB247, AB294, AB304, AB347) revealed more than fourfold reduction in TGC MICs (Table 2), and all five strains had *adeA* overexpression, and similarities in *adeA* sequences was 100% in 4 of the isolates. The similarity in *adeA* sequence of AB099 was 99.8%, with only a single amino-acid substitution (N295T). However, no significant differences were found among the responses of strains to anti-rAdeK antisera regarding their sequence homology (Table 3), gene expression level (Table 2) and binding ability of antisera to bacterial cells (Figure 3C). Furthermore, we examined the synergistic effect of anti-AdeK serum and four antibiotics including amikacin, meropenem, colistin and ampicillin/sulbactam, remained active against different portions of *A. baumannii* isolates. We found that only two isolates demonstrated a twofold reduction in the ampicillin-sulbactam MIC value from 64/32 to 32/16 μg/mL in the presence of anti-AdeK (Supplementary Table S3).

**TABLE 2:** Minimum inhibitory concentrations (MICs) of clinical isolates and synergistic effects of antigen-specific antisera on tigecycline (TGC) MICs^a^.

**Strains**	**Original**	**rAdeA**		**rAdeI**		**rAdeK**		**rTolC**	
	**TGC MIC**	**OE^*b*^**	**MIC**	**OE**	**MIC**	**OE**	**MIC**	**OE**	**MIC**
AB099	8	+	8	+	4	+	**2**	+	4
AB113	16	+	16	+	16	+	8	+	16
AB118	8	+	8	+	16	*−*	4	*−*	8
AB247	16	+	16	+	**4**	+	**2**	*−*	8
AB263	16	+	16	+	16	+	8	*−*	16
AB292	8	+	16	+	16	*−*	16	+	16
AB294	8	+	4	+	4	+	**2**	*−*	8
AB299	8	+	16	+	8	+	8	*−*	16
AB304	16	+	8	*−*	8	+	**4**	+	16
AB315	16	+	8	+	16	*−*	8	+	16
AB317	16	+	16	+	16	+	8	+	16
AB318	16	+	16	+	16	+	16	*−*	16
AB332	16	+	8	+	16	*−*	8	+	16
AB347	16	+	16	+	16	+	**4**	+	**4**
AB349	16	+	16	+	16	*−*	8	*−*	16

**^*a*^Bold font indicates a fourfold reduction in MIC value. Experiments were performed in triplicate and repeated three times, with similar results. ^*b*^OE: overexpression, gene expression level ≥ twofold compared to that of the ATCC17978 reference strain was considered significant overexpression;* +, *Yes; −, No.**

**TABLE 3:** Comparisons of efflux pump protein sequence similarities to *A. baumannii* ATCC 17978.

**Strain ID**	***adeA***	***adeI***	***adeK***	***tolC***
AB099	100%	100%	99.80% (N295T)	99.60% (Q85H, K180T)
AB113	99.30% (T138A)	100%	99.80% (G212L)	100%
AB118	99.30% (T138A)	100%	100%	100%
AB247	99.30% (T138A)	100%	100%	100%
AB263	99.30% (T138A)	100%	99.80% (G212C)	100%
AB292	99.30% (T138A)	100%	100%	100%
AB294	99.30% (T138A)	100%	100%	99.60% (E219V, Y220F)
AB299	99.30% (T138A)	100%	100%	100%
AB304	99.30% (T138A)	100%	100%	100%
AB315	99.30% (T138A)	100%	99.80% (L296S)	100%
AB317	99.30% (T138A)	100%	99.80% (Q193H)	99.10% (T293I, R325G, T326S, S327Y)
AB318	99.30% (T138A)	100%	100%	100%
AB332	99.30% (T138A)	100%	100%	100%
AB347	99.30% (T138A)	100%	100%	98.20% (Q163R, N193C, Q195H, Y196D, A201T, A205E, N207D, A213T)
AB349	99.30% (T138A)	100%	100%	100%

### TGC Activity Potentiation by rAdeK Immunization in a Mouse Pneumonia Model

**FIGURE 4:**
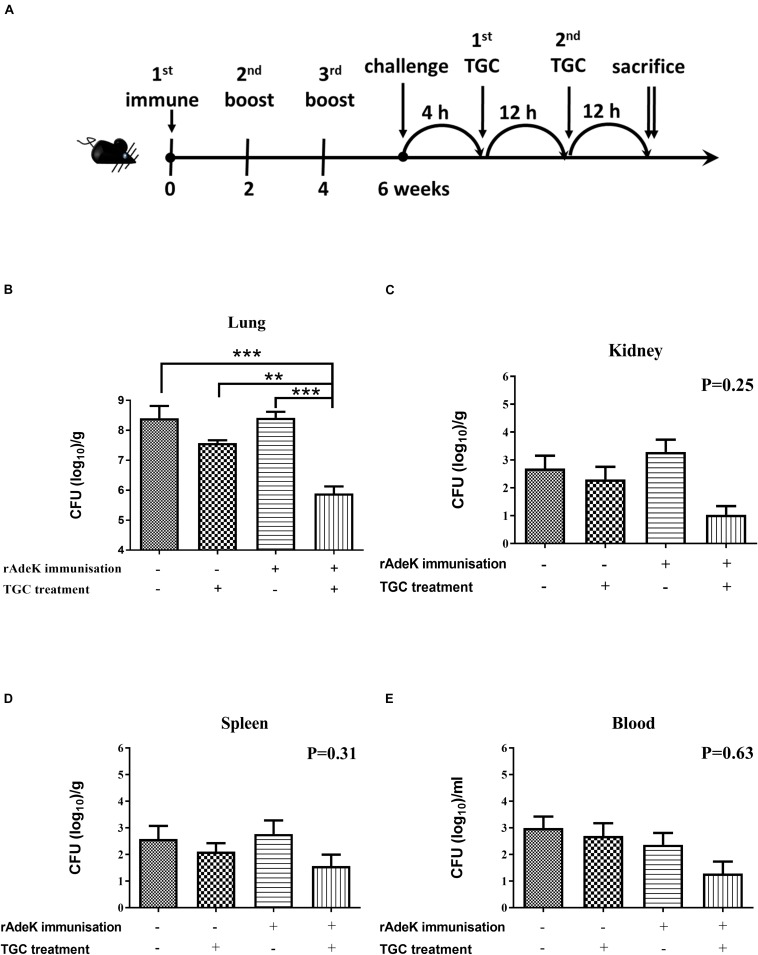
The efficacy of rAdeK immunization in a mouse pneumonia model. **(A)** Rational design of the animal model. Treatment was initiated 4 h after challenge against *A. baumannii* AB247 [tigecycline (TGC) minimum inhibitory concentration of 16 μg/mL], twice a day by subcutaneous injections. Bacterial loads were determined in different organs after the mice were sacrificed and were compared among the four groups. **(B–E)** Bacterial load in the mice lungs **(B)**, kidney **(C)**, spleen **(D)**, and blood **(E)** after 24 h TGC treatment compared among the four groups studied. The bars indicate the means ± the SEM. **p < 0.01; ***p < 0.001.

**FIGURE 5:**
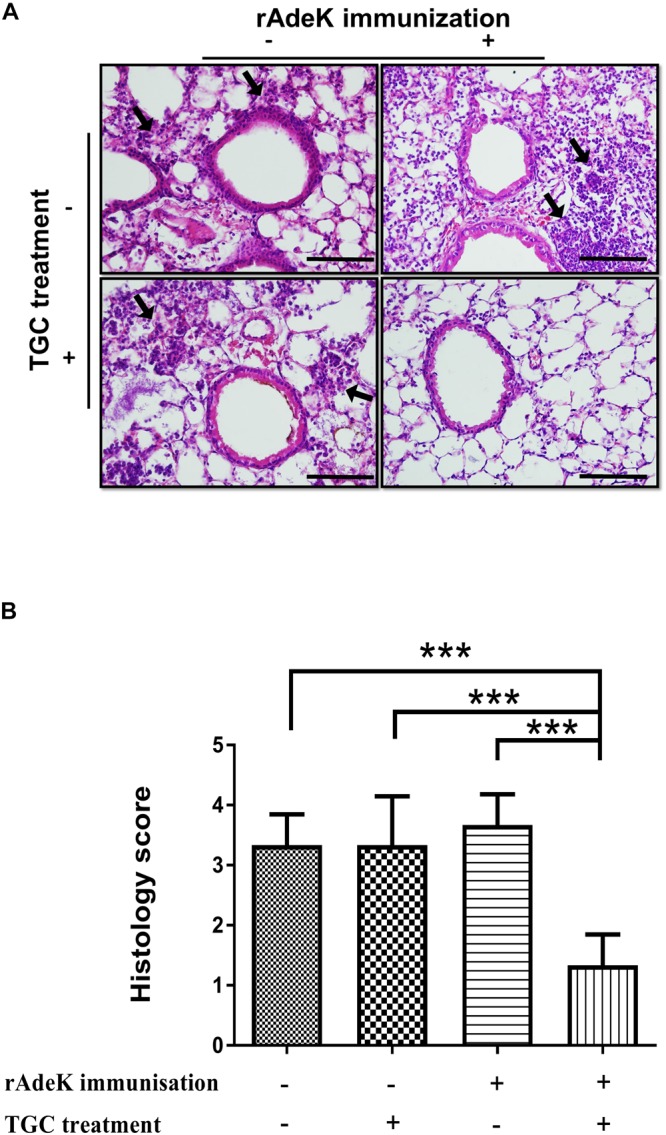
The histology of mouse lung sections in pneumonia model. **(A)** Hematoxylin and eosin staining of mouse lung sections obtained from each group. Images were taken at 40× by light microscopy and represented sections from three mice per group. The arrows indicate regions of peri-bronchovascular infiltration. Bars = 100 μm. **(B)** Lung inflammation was scored, the bars indicate the means of at least three samples ± SEM. ***p < 0.001.

According to the *in vitro* data, rAdeK antisera showed the highest capability to potentiate the effect of TGC against TGC-R *A. baumannii*, and also had an addition role to in antibody mediated killing, so rAdeK was selected as a vaccine candidate. The protocol for murine pneumonia model is shown in Figure 4A. An intratracheal challenge with a clinical isolate of AB247 was performed. AB247 was selected because of its high TGC-resistant phenotype (MIC 16 mg/L) and its significant response in broth microdilution evaluation. The results showed that TGC administration in rAdeK-immunized mice significantly reduced the bacterial load in the lungs compared to that in mice without immunization (Figure 4B, *p* < 0.001). The bacterial load was also reduced in the kidney, spleen, and blood, but without statistical significance (Figures 4C–E). The histopathology of lung tissues showed that neutrophil infiltration in the peri-bronchial vasculature region was lower in rAdeK-immunization and TGC-treated mice (Figure 5) compared to that in other groups. More importantly, there was no evidence of morbidity or mortality among mice during the immunization course, which suggested that rAdeK is safe and suitable for immunization.

## Discussion

**FIGURE 6:**
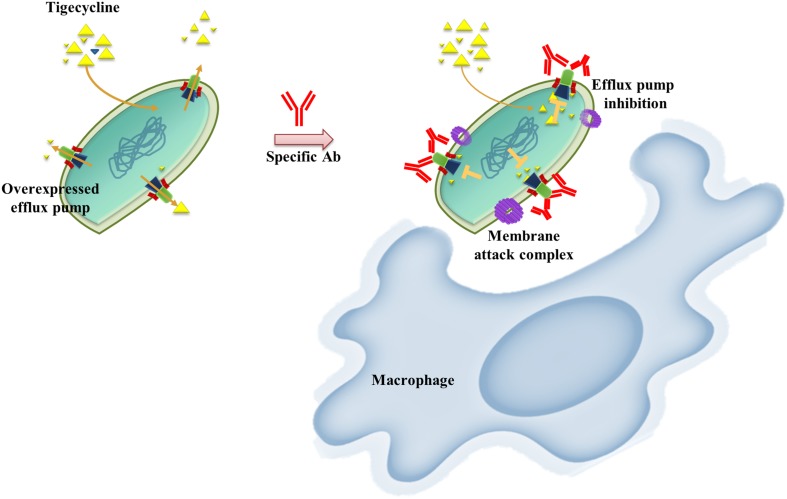
Schematic representation of the proposed mechanism of anti-efflux antibodies-mediated tigecycline efflux inhibition and bactericidal activity. Under overexpressed conditions, activated efflux pumps efficiently remove tigecycline to attenuate antibiotic activity **(left)**. These phenomena might be restored by using an anti-rAdeK antibody as an efflux pump inhibitor. Bactericidal effects might also be induced via complement-mediated lytic membrane attack complex pores and opsonophagocytic activity **(right)**.

AMR bacterial infection have been increasing dramatically in recent decades and account for about 80% of all severe bacterial infections (Du et al., 2018). Resistant phenotypes can arise from overexpression of intrinsic efflux activity to effectively respond to antibiotic or toxin related challenges (Du et al., 2018). Inhibition of antimicrobial determinants, such as efflux pump inhibitors (EPIs) is a feasible approach to preserve and improve the clinical performance of antituberculosis agents and overcome crucial drug-resistance challenges (Song and Wu, 2016). The use of EPIs could facilitate the revival of antibiotics and could be suitable for clinical applications. Unfortunately, no EPIs are yet available for clinical use (Abdali et al., 2017). We developed an alternative vaccine strategy targeting an antibiotic efflux pump. We tested this idea in TGC-resistant *A. baumannii*. We found that immunization with the RND efflux pump outer membrane protein, AdeK, interfered with the efflux activity of bacteria and, also induced antibodies with bactericidal effects (Figure 6). When co-administrated with TGC therapy, this strategy efficiently attenuated TGC-R *A. baumannii* pneumonia infection. A recent study showed that the combined treatment of anti-outer membrane vesicle serum and antibiotics could increase the intracellular aggregation of antibiotics by affecting porin function (Huang et al., 2019). The strategy significantly improved the antibiotic susceptibility of drug-resistant *A. baumannii* and strengthened the feasibility of combination therapy with vaccine and antibiotics. Accordingly, predominant resistance determinants with high homology, such as MecA, that confer resistance to methicillin in *Staphylococcus aureus* might also be good candidates for this approach.

Efflux-mediated TGC resistance has been extensively investigated in *A. baumannii*, especially in RND efflux pumps such as AdeABC, AdeFGH, AdeIJK, and AcrAB-TolC, in which overexpression leads to a TGC-resistant phenotype (Sugawara and Nikaido, 2014). Moreover, mutations in the *adeR* and *adeS* regulatory genes have been detected in TGC-R clinical isolates with efflux pump overexpression (Sun et al., 2014). Therefore, inhibition of efflux activity is a promising approach to restore TGC susceptibility. RND efflux systems usually comprise three different components assembling into a functional complex, including outer membrane protein, middle periplasmic protein and inner membrane protein (Du et al., 2018). AdeA and AdeI belonged to the inner membrane protein of the system, whereas AdeK and TolC belonged to the outer membrane protein of the system. The results of kinetic accumulation study in the present study showed that all the antisera could potentially attenuate bisbenzimide H33342 efflux activity in ATCC strains, indicating H33342 is the substrate of these efflux systems. Decreased accumulation of H33342 after 40 min of antisera treatment (Figure 3A) might indicate the activation of the redundant efflux system to extrude H33342. Compared to other antisera, rAdeK antisera efficiently reduced TGC MIC levels in the clinical isolates; this result might indicate the major contribution of AdeIJK in TGC resistance in *A. baumannii* (Rosenfeld et al., 2012). It is interesting that when targeting the same RND system (AdeIJK), different components of the system could have different effects. For example, antisera against AdeK could potentiate TGC effects but antisera against *Ade*I could not. This result indicated that the outer membrane component might be a better vaccine candidate than inner membrane protein when combined with antibiotic use. As demonstrated in Figure 3C, antisera against rAdeK derived from ATCC17978 could cross-react with all fifteen clinical isolates by flow cytometry assay. The *in vivo* study also demonstrated a good response in AdeK-vaccinated and TGC-treated mice, as they demonstrated less lung inflammation and reduced bacterial load in the lung. The bacteria load in other tissues was also lower in the AdeK-vaccinated and TGC-treated mice, but the difference was not significant. This might be due to the lower bacterial load (about 10^3^ CFU/g) in the no treatment or single treatment arms (rAdeK vaccinated or TGC-treated group), as *A. baumannii* is a bacterium with low virulence.

In addition to the potentiation of antibiotic treatment, the antisera had other roles as it could enhance the antibody mediated killing of bacteria. In the *in vivo* study, the effect of AdeK immunization might come from two aspects, one is antagonizing the efflux pump against antibiotic extrusion, another is through antibody mediated killing. It is unknown how the antisera of the efflux pump could reverse the antibiotic resistance. It is reported that antibodies may affect the function of specific antigens via conformation changes (Roguin and Retegui, 2003), or that this effect could be a result of blocking the channel of antibiotic extrusion. Some efflux pumps are associated with bacterial virulence and biofilm formation, which are responsible for host cell adhesion and invasion (Du et al., 2018). For example, TolC is a virulence factor associated with toxin translocation in *E. coli* (Lee et al., 2012). In our study, antisera derived from immunization with rTolC conferred significant complement-dependent bactericidal and opsonophagocytic activity. It is worth to determine whether rTolC could be an ideal vaccine candidate in future studies.

The non-responding strains also had similar *adeK* sequence and overexpression level of the gene. Unknown mechanisms for TGC resistance may have been present in the non-responding strains. Li et al. (2016) identified > 50 possible drug efflux pumps that could contribute to multidrug resistance from over 1000 genomes of *A. baumannii* strains. In addition, the antibody might need to be optimized to more effectively block the most critical epitope of the efflux protein. Future research should focus on physical data in three-dimensional structures of efflux pumps and specific antibodies need to be elucidated to understand the structure-function relationships in these pumps (Roguin and Retegui, 2003). Although only 26.7% of strains had reduced TGC MIC after adding antisera, low coverage of the bacteria population (even 1–4%) by immunization with resistant determinants might still be effective in eradicating the resistant population (Niewiadomska et al., 2019).

We examined the effect of the anti-AdeK antisera on amikacin, meropenem, colistin, and ampicillin/sulbactam (Supplementary Table S3). These results showed no significant synergistic effect to reverse the resistance against these antibiotics. These was not unexpected, as the major resistant mechanisms of these antibiotics are not through efflux pump. Instead, the major resistance mechanism for amikacin, meropenem and ampicillin/sulbactam is the production of antibiotic modifying or degrading enzymes (Lee et al., 2017), whereas modification or loss of lipopolysaccharide confers colistin resistance in *A. baumannii*.

It is important to note that efflux pumps are conserved not only in *A. baumannii* but also in different bacterial genera. The AdeK protein has sequence homology to efflux proteins in other nosocomial “bad bugs” listed by the Infectious Diseases Society of America (Boucher et al., 2009; Supplementary Table S4), thus having the potential of broader coverage and application in the near future. A recent report also supported that AdeK and other 24 resistant determinants are predicted as vaccine candidates to strengthen antibiotic treatments (Ni et al., 2017).

## Conclusion

In conclusion, our results demonstrate that active immunization with antibiotic-resistant determinants may be a promising approach to combat multidrug-resistant pathogens in high-risk population.

## Data Availability Statement

The raw data supporting the conclusions of this article will be made available by the authors, without undue reservation, to any qualified researcher.

## Ethics Statement

The animal study was reviewed and approved by National Defense Medical Center Institutional Animal Care and Use Committee (NDMC IACUC-17-206).

## Author Contributions

M-HC, T-LC, and Y-PC contributed to the conception and design of the studies. M-HC, Y-SY, S-CK, and Y-PC contributed to the execution of the animal vaccination studies. M-HC, Y-TL, and J-RS carried out the laboratory *in vitro* assays. M-HC, Y-SY, Y-CW, S-CK, Y-TL, Y-PC, and T-LC were involved in the drafting, revision, and approval of the final version of the manuscript.

## Conflict of Interest

The authors declare that the research was conducted in the absence of any commercial or financial relationships that could be construed as a potential conflict of interest.

## References

[B1] Abdali N. Parks J. M. Haynes K. M. Chaney J. L. Green A. T. Wolloscheck D. (2017). Reviving antibiotics: efflux pump inhibitors that interact with AcrA, a membrane fusion protein of the AcrAB-TolC multidrug efflux pump. *ACS Infect. Dis.* 3 89–98. 10.1021/acsinfecdis.6b00167 27768847 PMC5553321

[B2] Alekshun M. N. Levy S. B. (2007). Molecular mechanisms of antibacterial multidrug resistance. *Cell* 128 1037–1050. 10.1016/j.cell.2007.03.004 17382878

[B3] Ardehali S. H. Azimi T. Fallah F. Owrang M. Aghamohammadi N. Azimi L. (2019). Role of efflux pumps in reduced susceptibility to tigecycline in *Acinetobacter baumannii*. *New Microbes. New Infect.* 30:100547. 10.1016/j.nmni.2019.100547 31193724 PMC6541740

[B4] Boucher H. W. Talbot G. H. Bradley J. S. Edwards J. E. Gilbert D. Rice L. B. (2009). Bad bugs, no drugs: no ESKAPE! an update from the infectious diseases society of America. *Clin. Infect. Dis.* 48 1–12. 10.1086/595011 19035777

[B5] Chiang M. H. Sung W. C. Lien S. P. Chen Y. Z. Lo A. F. Huang J. H. (2015). Identification of novel vaccine candidates against *Acinetobacter baumannii* using reverse vaccinology. *Hum. Vaccin. Immunother.* 11 1065–1073. 10.1080/21645515.2015.1010910 25751377 PMC4514290

[B6] CLSI (2017). *M100-S27: Performance Standards for Antimicrobial Susceptibility Testing: 27th Informational Supplement.* Wayne, PA: Clinical and Laboratory Standards Institute.

[B7] Du D. Wang-Kan X. Neuberger A. Van Veen H. W. Pos K. M. Piddock L. J. V. (2018). Multidrug efflux pumps: structure, function and regulation. *Nat. Rev. Microbiol.* 16 523–539. 30002505 10.1038/s41579-018-0048-6

[B8] Gagneux-Brunon A. Lucht F. Launay O. Berthelot P. Botelho-Nevers E. (2018). Vaccines for healthcare-associated infections: present, future, and expectations. *Expert Rev. Vaccin.* 17 421–433. 10.1080/14760584.2018.1470507 29697286

[B9] Garcia-Quintanilla M. Pulido M. R. Carretero-Ledesma M. Mcconnell M. J. (2016). Vaccines for antibiotic-resistant bacteria: possibility or pipe dream? *Trends Pharmacol. Sci.* 37 143–152. 10.1016/j.tips.2015.10.003 26574183

[B10] Huang W. Yao Y. Long Q. Yang X. Sun W. Liu C. (2014). Immunization against multidrug-resistant *Acinetobacter baumannii* effectively protects mice in both pneumonia and sepsis models. *PLoS One* 9:e100727. 10.1371/journal.pone.0100727 24956279 PMC4067354

[B11] Huang W. Zhang Q. Li W. Chen Y. Shu C. Li Q. (2019). Anti-outer membrane vesicle antibodies increase antibiotic sensitivity of pan-drug-resistant *Acinetobacter baumannii*. *Front. Microbiol.* 10:1379. 10.3389/fmicb.2019.01379 31275290 PMC6591364

[B12] Imai K. Asakawa N. Tsuji T. Akazawa F. Ino A. Sonoyama M. (2008). SOSUI-GramN: high performance prediction for sub-cellular localization of proteins in gram-negative bacteria. *Bioinformation* 2 417–421. 10.6026/97320630002417 18795116 PMC2533062

[B13] Jansen K. U. Knirsch C. Anderson A. S. (2018). The role of vaccines in preventing bacterial antimicrobial resistance. *Nat. Med.* 24 10–19. 10.1038/nm.4465 29315295

[B14] Jia B. Raphenya A. R. Alcock B. Waglechner N. Guo P. Tsang K. K. (2017). CARD 2017: expansion and model-centric curation of the comprehensive antibiotic resistance database. *Nucleic Acids Res.* 45 D566–D573. 10.1093/nar/gkw1004 27789705 PMC5210516

[B15] Joice R. Lipsitch M. (2013). Targeting imperfect vaccines against drug-resistance determinants: a strategy for countering the rise of drug resistance. *PLoS One* 8:e68940. 10.1371/journal.pone.0068940 23935910 PMC3723804

[B16] Kumar S. Stecher G. Tamura K. (2016). MEGA7: molecular evolutionary genetics analysis version 7.0 *for bigger datasets*. *Mol. Biol. Evol.* 33 1870–1874. 10.1093/molbev/msw054 27004904 PMC8210823

[B17] Laure D. Virginie P. Alexandr N. Lenie D. Sylvain B. (2010). The population structure of *Acinetobacter baumannii*: expanding multiresistant clones from an ancestral susceptible genetic pool. *PLoS One* 5:e10034. 10.1371/journal.pone.0010034 20383326 PMC2850921

[B18] Lee C. R. Lee J. H. Park M. Park K. S. Bae I. K. Kim Y. B. (2017). Biology of *Acinetobacter baumannii*: pathogenesis, antibiotic resistance mechanisms, and prospective treatment options. *Front. Cell Infect. Microbiol.* 7:55. 10.3389/fcimb.2017.00055 28348979 PMC5346588

[B19] Lee M. Jun S. Y. Yoon B. Y. Song S. Lee K. Ha N. C. (2012). Membrane fusion proteins of type I secretion system and tripartite efflux pumps share a binding motif for TolC in gram-negative bacteria. *PLoS One* 7:e40460. 10.1371/journal.pone.0040460 22792337 PMC3391258

[B20] Li L. Hassan K. A. Brown M. H. Paulsen I. T. (2016). Rapid multiplexed phenotypic screening identifies drug resistance functions for three novel efflux pumps in *Acinetobacter baumannii*. *J. Antimicrob. Chemother.* 71 1223–1232. 10.1093/jac/dkv460 26832750

[B21] McConnell M. J. Domínguez-Herrera J. Smani Y. López-Rojas R. Docobo-Pérez F. Pachón J. (2011). Vaccination with outer membrane complexes elicits rapid protective immunity to multidrug-resistant *Acinetobacter baumannii*. *Infect. Immun.* 79 518–526. 10.1128/IAI.00741-10 20974823 PMC3019872

[B22] Ni W. Han Y. Zhao J. Wei C. Cui J. Wang R. (2016). Tigecycline treatment experience against multidrug-resistant *Acinetobacter baumannii* infections: a systematic review and meta-analysis. *Int. J. Antimicrob. Agents* 47 107–116. 10.1016/j.ijantimicag.2015.11.011 26742726

[B23] Ni Z. Chen Y. Ong E. He Y. (2017). Antibiotic resistance determinant-focused *Acinetobacter baumannii* vaccine designed using reverse vaccinology. *Int. J. Mol. Sci.* 18:458. 10.3390/ijms18020458 28230771 PMC5343991

[B24] Niewiadomska A. M. Jayabalasingham B. Seidman J. C. Willem L. Grenfell B. Spiro D. (2019). Population-level mathematical modeling of antimicrobial resistance: a systematic review. *BMC Med.* 17:81. 10.1186/s12916-019-1314-9 31014341 PMC6480522

[B25] Nordmann P. Poirel L. (2019). Epidemiology and diagnostics of carbapenem resistance in gram-negative bacteria. *Clin. Infect. Dis.* 69 S521–S528. 10.1093/cid/ciz824 31724045 PMC6853758

[B26] Noto M. J. Becker K. W. Boyd K. L. Schmidt A. M. Skaar E. P. (2017). RAGE-mediated suppression of interleukin-10 results in enhanced mortality in a murine model of *Acinetobacter baumannii* sepsis. *Infect. Immun.* 85 e954–e916. 10.1128/IAI.00954-16 28052995 PMC5328494

[B27] Perez F. Bonomo R. A. (2014). Vaccines for *Acinetobacter baumannii*: thinking “out of the box”. *Vaccine* 32 2537–2539. 10.1016/j.vaccine.2014.03.031 24662709 PMC4028134

[B28] Pichardo C. Pachon-Ibanez M. E. Docobo-Perez F. Lopez-Rojas R. Jimenez-Mejias M. E. Garcia-Curiel A. (2010). Efficacy of tigecycline vs. *imipenem in the treatment of experimental Acinetobacter baumannii* murine pneumonia. *Eur. J. Clin. Microbiol. Infect. Dis.* 29 527–531. 10.1007/s10096-010-0890-6 20182760

[B29] Pillar C. M. Draghi D. C. Dowzicky M. J. Sahm D. F. (2008). In vitro activity of tigecycline against gram-positive and gram-negative pathogens as evaluated by broth microdilution and Etest. *J. Clin. Microbiol.* 46 2862–2867. 10.1128/JCM.00637-08 18596149 PMC2546738

[B30] Rappuoli R. Black S. Bloom D. E. (2019). Vaccines and global health: in search of a sustainable model for vaccine development and delivery. *Sci. Transl. Med.* 11:eaaw2888. 10.1126/scitranslmed.aaw2888 31217336

[B31] Richmond G. E. Chua K. L. Piddock L. J. (2013). Efflux in *Acinetobacter baumannii* can be determined by measuring accumulation of H33342 (bis-benzamide). *J. Antimicrob. Chemother.* 68 1594–1600. 10.1093/jac/dkt052 23467176 PMC3682688

[B32] Roguin L. P. Retegui L. A. (2003). Monoclonal antibodies inducing conformational changes on the antigen molecule. *Scand. J. Immunol.* 58 387–394. 10.1046/j.1365-3083.2003.01320.x 14507303

[B33] Rosenfeld N. Bouchier C. Courvalin P. Perichon B. (2012). Expression of the resistance-nodulation-cell division pump AdeIJK in *Acinetobacter baumannii* is regulated by AdeN, a TetR-type regulator. *Antimicrob. Agents Chemother.* 56 2504–2510. 10.1128/AAC.06422-11 22371895 PMC3346617

[B34] Song L. Wu X. (2016). Development of efflux pump inhibitors in antituberculosis therapy. *Int. J. Antimicrob. Agents* 47 421–429. 10.1016/j.ijantimicag.2016.04.007 27211826

[B35] Sugawara E. Nikaido H. (2014). Properties of AdeABC and AdeIJK efflux systems of *Acinetobacter baumannii* compared with those of the AcrAB-TolC system of *Escherichia coli*. *Antimicrob. Agents Chemother.* 58 7250–7257. 10.1128/AAC.03728-14 25246403 PMC4249520

[B36] Sun J. R. Perng C. L. Lin J. C. Yang Y. S. Chan M. C. Chang T. Y. (2014). AdeRS combination codes differentiate the response to efflux pump inhibitors in tigecycline-resistant isolates of extensively drug-resistant *Acinetobacter baumannii*. *Eur. J. Clin. Microbiol. Infect. Dis.* 33 2141–2147. 10.1007/s10096-014-2179-7 24939621

[B37] Sun Y. Cai Y. Liu X. Bai N. Liang B. Wang R. (2013). The emergence of clinical resistance to tigecycline. *Int. J. Antimicrob. Agents* 41 110–116. 10.1016/j.ijantimicag.2012.09.005 23127485

[B38] Uchiyama I. Mihara M. Nishide H. Chiba H. (2014). MBGD update 2015: microbial genome database for flexible ortholog analysis utilizing a diverse set of genomic data. *Nucleic Acids Res.* 43 D270–D276. 10.1093/nar/gku1152 25398900 PMC4383954

[B39] Yu C. S. Cheng C. W. Su W. C. Chang K. C. Huang S. W. Hwang J. K. (2014). CELLO2GO: a web server for protein subCELlular LOcalization prediction with functional gene ontology annotation. *PLoS One* 9:e99368. 10.1371/journal.pone.0099368 24911789 PMC4049835

[B40] Yu N. Y. Wagner J. R. Laird M. R. Melli G. Rey S. Lo R. (2010). PSORTb 3.0: improved protein subcellular localization prediction with refined localization subcategories and predictive capabilities for all prokaryotes. *Bioinformatics (Oxford, England)* 26 1608–1615. 10.1093/bioinformatics/btq249 20472543 PMC2887053

