# Supplementary material for: Confronting Tigecycline-Resistant Acinetobacter baumannii via Immunization Against Conserved Resistance Determinants
Source: Front Microbiol. 2020 Mar 31;11:536. doi: 10.3389/fmicb.2020.00536 (PMC7136391; doi:10.3389/fmicb.2020.00536)
Supplement: Supplementary file 1 [file Data_Sheet_1.docx]

| **Table S1. The profiles of the *Acinetobacter baumannii* genomes from the NCBI used in this study** | | | | | | | | | | | | | |
| --- | --- | --- | --- | --- | --- | --- | --- | --- | --- | --- | --- | --- | --- |
| **Strain** | **CDSs** | **Country** | **Accession no.** | AMK | SAM | FEP | CIP | CAZ | CST | LVX | MEM | TZP | TET |
| ATCC17978 | 3,993 | USA | NC_009085 | S | S | S | S | S | S | S | S | S | S |
| AB307-0294 | 3,550 | USA | NC_011595 | S | S | S | S | NA | NA | NA | S | S | S |
| AYE | 3,913 | France | NC_010410 | R | NA | R | R | R | S | I | S | S | R |
| AB0057 | 3,969 | USA | NC_011586 | S | R | R | R | NA | NA | NA | R | R | S |
| ACICU | 3,908 | Italy | NC_010611 | R | NA | R | R | NA | R | NA | R | R | NA |
| MDR-ZJ06 | 3,965 | China | NC_017171 | R | R | R | R | R | S | NA | R | R | R |
| D1279779 | 3,396 | Australia | NC_020547 | NA | NA | NA | NA | NA | S | NA | S | NA | S |
| 1656-2 | 3,925 | Korea | NC_017162 | R | R | R | R | R | NA | NA | NA | R | NA |
| TCDC-AB0715 | 4,136 | Taiwan | NC_017387 | NA | NA | R | R | NA | NA | R | R | NA | NA |
| MDR-TJ | 3,936 | China | NC_017847 | R | NA | R | R | R | NA | R | R | R | NA |
| TYTH-1 | 3,682 | Taiwan | NC_018706 | S | R | NA | S | R | NA | R | R | R | NA |
| BJAB07104 | 3,861 | China | NC_021726 | R | NA | R | R | R | S | R | R | R | NA |
| BJAB0868 | 3,819 | China | NC_021729 | R | NA | R | R | R | S | R | R | R | NA |
| BJAB0715 | 3,908 | China | NC_021733 | R | NA | R | R | R | S | R | R | R | NA |
| ZW85-1 | 3,465 | China | NC_023028 | NA | NA | NA | NA | NA | R | NA | NA | NA | NA |
| Abbreviation: CDSs, coding sequences; AMK, amikacin; SAM, ampicillin-sulbactam; FEP, cefepime; CIP, ciprofloxacin; CAZ, ceftazidime; CST, colistin; LVX, levofloxacin; MEM, meropenem; TZP, piperacillin-tazobactam; TET, tetracycline; S, susceptible; I, intermediate; R, resistant; NA, non-available | | | | | | | | | | | | | |

**Supplementary results**

| Table S2. Primers used for polymerase chain reaction (PCR) or quantitative real-time PCR (qRT-PCR) | | |
| --- | --- | --- |
| Primer | **Sequence (5′→3′)^a^** | **Reference** |
| A1S_0255_NdeI_F | GGATCC**CATATG**ATGGCAACAGGCGCTTTAC | This study for protein purification |
| A1S_0255_XhoI_R | TCCG**CTCGAG**CTGATACGTTAACCAGCTGTTT |  |
| A1S_1751_NdeI_F | GCATCC**CATATG**ATGTTGTTTGAAGATATTAATGTTGAC |  |
| A1S_1751_XhoI_R | TCCG**CTCGAG**GTGGTGCCATGGTGGTTGCGCCCCCTCA |  |
| A1S_2735_NdeI_F | GGATCC**CATATG**ATGCCGCCTGCAGAAGTA |  |
| A1S_2735_XhoI_R | TCCG**CTCGAG**TGCATTTGAAGCTGCTTTCTGT |  |
| A1S_2737_NcoI_F | CATG**CCATGG**atgcgcggcccagaa |  |
| A1S_2737_XhoI_R | TCCG**CTCGAG**ttgctttttaagttcagcactaga |  |
| RT_rpoB_F | TCCGCACGTAAAGTAGGAAC | (Coyne et al., 2010) |
| RT_rpoB_R | ATGCCGCCTGAAAAAGTAAC |  |
| RT_adeA_F | TTGATCGTGCTTCTATTCCTCAAG |  |
| RT_adeA_R | GGCTCGCCACTGATATTACGTT |  |
| RT_adeI_F | CAAATGCAAATGTAGATCTTGG |  |
| RT_adeI_R | AAACTGCCTTTACTTAGTTG |  |
| RT_adeK_F | TTGATAGTTACTTGACTGTTC |  |
| RT_adeK_R | GGTTGGTGAACCACTGTATC |  |
| RT_tolC_F | GCAGGCATGTTTTTGATTTTCATTTGTATT | (Srinivasan et al., 2015) |
| RT_tolC_R | AGCAAAACTCGACGATGTAAAACTCCATAA |  |
| A1S_0255_F | GTTTTGGTTGGCGAAAGTGT | This study for PCR identification |
| A1S_0255_R | AAGCCACCCATGGTCATAAA |  |
| A1S_1751_F | CGCGCTCAATTAAGGTTACA |  |
| A1S_1751_R | AGGGCGATACCAATACGATG |  |
| A1S_2735_F | TTGTGTGTATGGGGCTTTCA |  |
| A1S_2735_R | TTCAACTGTTTCAGCCGATG |  |
| A1S_2737_F | TTCGGTTTCGGTGTACTTCC |  |
| A1S_2737_R | AAGTCACGACCCACATGCTT |  |
| ^a^ Restriction sites are highlighted in bold and included in the primer name | | |

| **Table S3. Minimum inhibitory concentrations (µg/mL) values of clinical isolates and synergistic effects of rAdeK-specific antisera on different antibiotics^a^** | | | | | | | | | | | | |
| --- | --- | --- | --- | --- | --- | --- | --- | --- | --- | --- | --- | --- |
| Antibiotic  Isolates | MIC of amikacin | | | MIC of meropenem | | | MIC of colistin | | | MIC of ampicillin-sulbactam | | |
|  | Origin | PBS | anti-rAdeK | Origin | PBS | anti-rAdeK | Origin | PBS | anti-rAdeK | Origin | PBS | anti-rAdeK |
| Ab099 | >128 | >128 | >128 | >16 | >16 | >16 | <=1 | <=1 | <=1 | 16/8 | 32/16 | 32/16 |
| Ab113 | >128 | >128 | >128 | >16 | >16 | >16 | <=1 | <=1 | <=1 | 32/16 | 32/16 | 32/16 |
| Ab118 | >128 | >128 | >128 | >16 | >16 | >16 | <=1 | <=1 | <=1 | 32/16 | 64/32 | 32/16 |
| Ab247 | >128 | >128 | >128 | >16 | >16 | >16 | <=1 | <=1 | <=1 | >=64/32 | >=64/32 | >=64/32 |
| Ab263 | >128 | >128 | >128 | >16 | >16 | >16 | <=1 | <=1 | <=1 | 64/32 | >=64/32 | **32/16** |
| Ab292 | >128 | >128 | >128 | >16 | >16 | >16 | <=1 | <=1 | <=1 | 32/16 | 32/16 | 32/16 |
| Ab294 | >128 | >128 | >128 | >16 | >16 | >16 | <=1 | <=1 | <=1 | 64/32 | 64/32 | 64/32 |
| Ab299 | >128 | >128 | >128 | >16 | >16 | >16 | <=1 | <=1 | <=1 | 64/32 | 64/32 | 64/32 |
| Ab304 | >128 | >128 | >128 | >16 | >16 | >16 | <=1 | <=1 | <=1 | 32/16 | 32/16 | 32/16 |
| Ab315 | >128 | >128 | >128 | >16 | >16 | >16 | <=1 | <=1 | <=1 | 32/16 | 32/16 | 32/16 |
| Ab317 | >128 | >128 | >128 | >16 | >16 | >16 | <=1 | <=1 | <=1 | 64/32 | 32/16 | 32/16 |
| Ab318 | >128 | >128 | >128 | >16 | >16 | >16 | <=1 | <=1 | <=1 | 64/32 | >=64/32 | 64/32 |
| Ab332 | >128 | >128 | >128 | >16 | >16 | >16 | <=1 | <=1 | <=1 | 64/32 | 64/32 | **32/16** |
| Ab347 | >128 | >128 | >128 | >16 | >16 | >16 | <=1 | <=1 | <=1 | 32/16 | 64/32 | 64/32 |
| Ab349 | >128 | >128 | >128 | >16 | >16 | >16 | <=1 | <=1 | <=1 | 64/32 | 64/32 | 64/32 |
| ^a^Bold font indicates a two-fold reduction in MIC value. Experiments were performed in triplicate and repeated three times, with similar results. | | | | | | | | | | | | |

| **Table S4. ESKAPE**^a^ **pathogen protein sequence similarities to *Acinetobacter baumannii*** **ATCC17978 AdeK** | | | | |
| --- | --- | --- | --- | --- |
| Pathogens | Homology gene | Accession number | Similarity^b^ | E value^b^ |
| *Enterobacteriaceae* | multidrug transporter | WP_009652383 | 41% | 6e-107 |
| *Staphylococcus aureus* | RND transporter | WP_111116190 | 29% | 1e-31 |
| *Klebsiella pneumoniae* | efflux transporter outer membrane subunit | WP_142483140. | 41% | 6e-103 |
| *Pseudomonas aeruginosa* | multidrug efflux RND transporter outer membrane channel subunit OprM | WP_004346588 | 43% | 5e-115 |
| *Escherichia coli* | efflux transporter outer membrane subunit | WP_053266434 | 44% | 4e-112 |
| ^a^ESKAPE, *Enterococcus faecium*, *Staphylococcus aureus*, *Klebsiella pneumoniae*, *Acinetobacter baumannii*, *Pseudomonas aeruginosa*, and *Enterobacter* species (Boucher et al., 2009)  ^b^Similarity and E-values were obtained from the Basic Local Alignment Search Tool (BLASTP) from the National Center For Biotechnology Information **(**NCBI) website. | | | | |

### References

Boucher, H.W., Talbot, G.H., Bradley, J.S., Edwards, J.E., Gilbert, D., Rice, L.B., Scheld, M., Spellberg, B., and Bartlett, J. (2009). Bad bugs, no drugs: no ESKAPE! An update from the Infectious Diseases Society of America. *Clin Infect Dis* 48**,** 1-12.

Coyne, S., Guigon, G., Courvalin, P., and Perichon, B. (2010). Screening and quantification of the expression of antibiotic resistance genes in Acinetobacter baumannii with a microarray. *Antimicrob Agents Chemother* 54**,** 333-340.

Kumar, S., Stecher, G., and Tamura, K. (2016). MEGA7: Molecular Evolutionary Genetics Analysis version 7.0 for bigger datasets. *Mol Biol Evol* 33**,** 1870-1874.

Srinivasan, V.B., Vaidyanathan, V., and Rajamohan, G. (2015). AbuO, a TolC-like outer membrane protein of Acinetobacter baumannii, is involved in antimicrobial and oxidative stress resistance. *Antimicrob Agents Chemother* 59**,** 1236-1245.
